# Mediators of the association between educational attainment and carpal tunnel syndrome: A 2-sample, 2-step Mendelian randomization study

**DOI:** 10.1097/MD.0000000000040302

**Published:** 2024-10-25

**Authors:** Yunrong Lai, Weiquan Hu, Suping Hu, Qinglin Liu, Xianping Huang, Qinfei Zhao

**Affiliations:** a Department of Imaging, Fifth People’s Hospital of Ganzhou, Ganzhou, Jiangxi, China; b Department of Joint Surgery, Ganzhou People’s Hospital, Ganzhou, Jiangxi, China; c Department of Emergency, First Affiliated Hospital of Gannan Medical University, Ganzhou, Jiangxi, China; d Department of Laboratory Medicine, First Affiliated Hospital of Gannan Medical University, Ganzhou, Jiangxi, China.

**Keywords:** carpal tunnel syndrome, educational attainment, multivariable Mendelian randomization, 2-step Mendelian randomization, univariable Mendelian randomization

## Abstract

To clarify the causal relationship and potential mediators between educational attainment and carpal tunnel syndrome (CTS), as well as to evaluate whether educational attainment, cognition, and intelligence independently exert causal effects on CTS, we employed univariable Mendelian randomization (MR), multivariable MR, reverse MR, and 2-step MR approaches. Our research demonstrates that educational attainment exerts an independent causal effect on CTS, with this causal relationship being unidirectional. Five mediators were identified as significant influencers within the causal pathways connecting educational attainment and CTS. Targeting these mediators may be beneficial in the prevention of CTS.

## 1. Introduction

Carpal tunnel syndrome (CTS) is a prevalent type of peripheral neuropathy resulting from median nerve compression of the wrist. It clinically manifest as pain and numbness in the hand and wrist, frequently progressing to weakness and atrophy of the thenar muscles.^[1]^ The conventional approach for diagnosing CTS involves a combination of clinical symptomatology, electromyography, and nerve conduction studies. The reported incidence of CTS varies considerably among studies. According to 1 survey, the prevalence of CTS is estimated to be 9.6%.^[2]^ Numerous studies have underscored its significance in occupational contexts, particularly among workers involved in repetitive fine motor tasks, prolonged wrist flexion, and frequent operation of power tools.^[2–4]^ CTS significantly affects physical health, quality of life, and work productivity, thereby imposing a substantial burden both on families and society. This necessitates careful attention from healthcare providers.

Occupational exposure, lifestyle habits, mental health, and various physical diseases are factors associated with educational attainment.^[5–7]^ Moreover, higher educational attainment has consistently been demonstrated to serve as a protective factor in numerous studies.^[8,9]^ Additionally, epidemiological research has established an inverse relationship between educational attainment and CTS incidence.^[10]^ It is important to note that residual, unmeasured confounding, or measurement inaccuracies are inherent in all observational studies. Randomized controlled trials (RCTs) are regarded as the universal standard for establishing causal connections. However, assessing the impact of educational attainment on CTS through RCTs may be both time-consuming and ethically challenging, as intervention in the educational process is typically viewed as unethical. Consequently, implementation of RCTs in this domain may pose considerable obstacles. An Mendelian randomization (MR) study that is less vulnerable to reverse causality and confounding variables would be ideal for investigating causal relationship in this context.

## 2. Materials and methods

### 2.1. Study design

**Figure 1.**
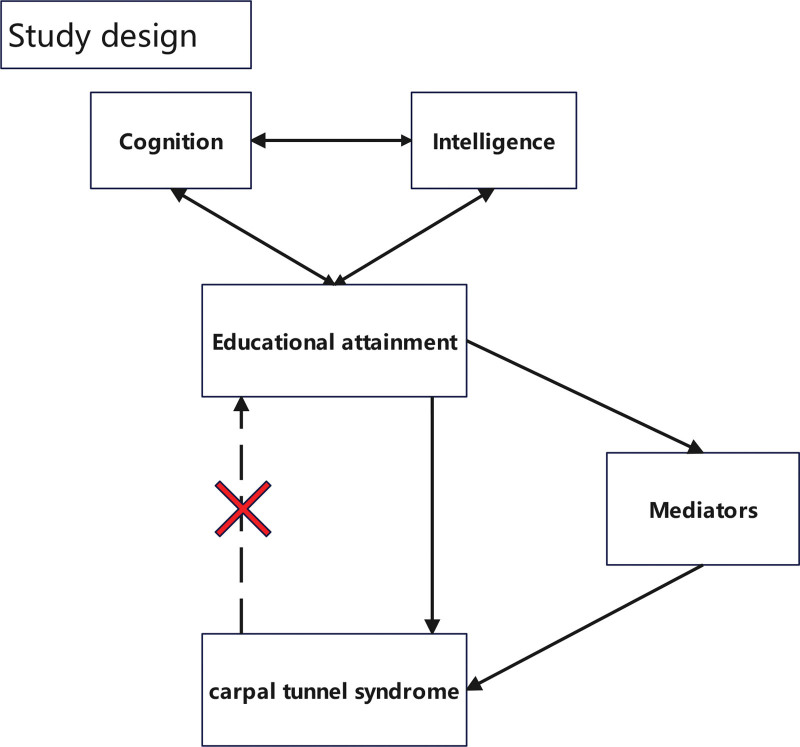
Overview of the study design.

**Figure 2.**
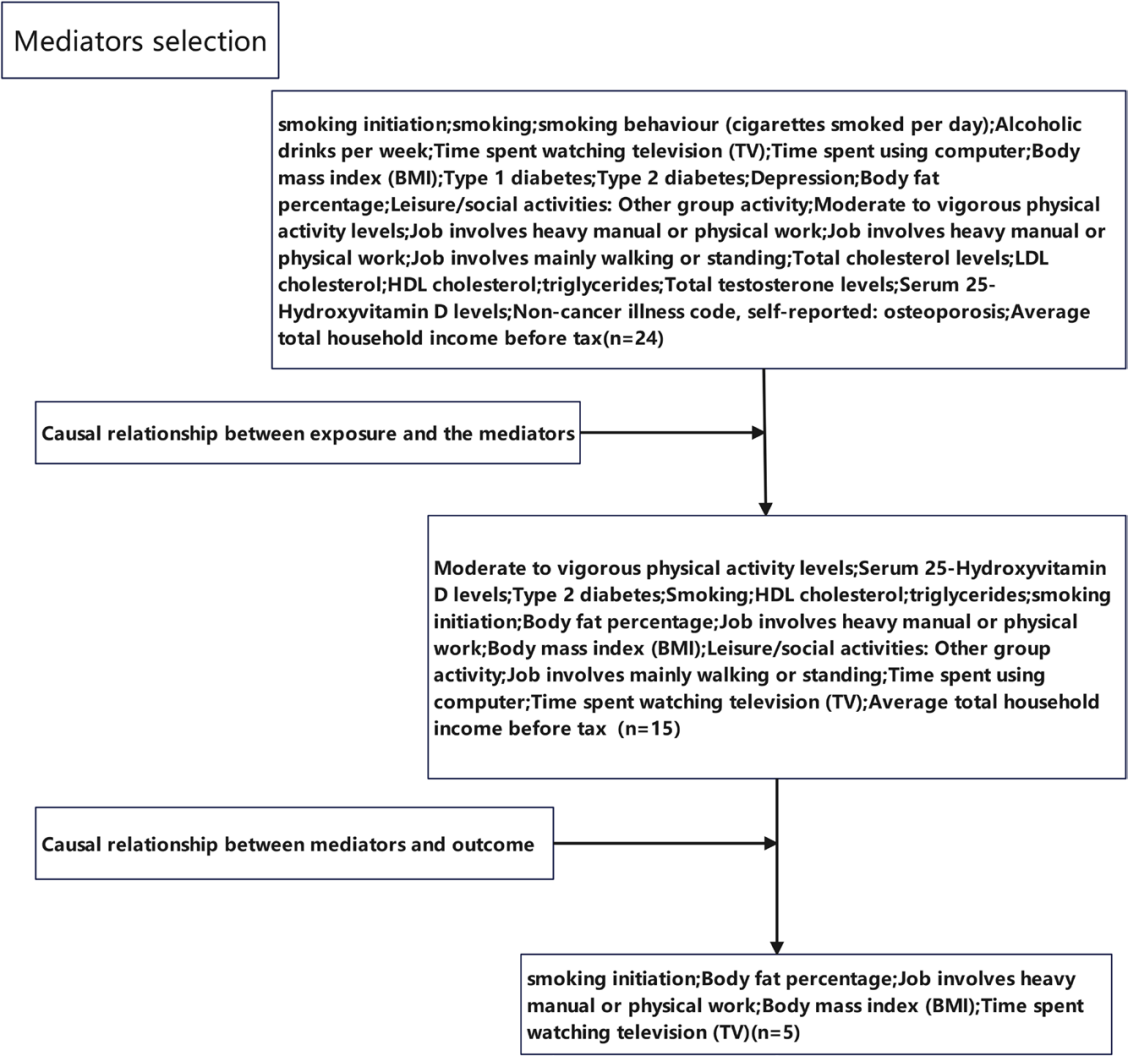
Mediators selection.

In this study, we assessed the causal interrelations among educational attainment, cognition, intelligence, and CTS using univariable MR (UVMR) and multivariable MR (MVMR) methodologies. Subsequently, we performed reverse MR analysis to confirm the unidirectional influence of educational attainment on CTS. Finally, we performed a 2-step MR analysis to explore whether health and lifestyle factors mediate this association. The initial step involved assessing the causal effect of educational attainment on health and lifestyle factors, followed by screening for health and lifestyle factors strongly associated with educational attainment. The second step involved assessing the causal effect of filtered health and lifestyle factors on CTS and calculating both the mediation effect and proportion. Figures 1 and 2 provide detailed overview of the study design and criteria for mediator selection.

### 2.2. Data sources

The dataset employed in this study was sourced from the Integrative Epidemiology Unit Open Genome-Wide Association Study website (https://gwasmrcieu.ac.uk). Specifically, data concerning educational attainment were provided by the Loh PR published in 2018.^[11]^ This dataset encompasses metrics, such as college completion (ebi-a-GCST90029012) and years of education (ebi-a-GCST90029013). The college completion dataset included a total of 470,941 samples alongside 11,972,619 single nucleotide polymorphisms (SNPs), whereas the years of education dataset comprised 461,457 samples with the same number of SNPs. Additionally, the CTS dataset (ebi-a-GCST90018813) derived from Sakaue S’s research published in 2021 included 480,201 samples and 24,181,062 SNPs.^[12]^ A comprehensive review of the existing literature suggests that certain diseases and life style factors may be associated with CTS incidence. Consequently, we identified several potential mediators, including: smoking initiation, smoking behavior (cigarettes smoked per day), smoking, duration of television (TV) viewing, alcoholic drinks per week, time spent on computer usage, body fat percentage, body mass index (BMI), depression, levels of moderate to vigorous physical activity, participation in leisure/social activities, type 1 diabetes, type 2 diabetes, jobs primarily requiring walking or standing, occupations involving heavy manual or physical labor, non-cancer illness codes, total cholesterol levels, high-density lipoprotein cholesterol, low-density lipoprotein cholesterol, self-reported osteoporosis, triglycerides, serum 25-hydroxyvitamin D levels, total testosterone levels, and average total household income prior to taxation. The GWAS contributing to this investigation were conducted on individuals of European descent, and all participants in the original GWAS provided written informed consent. Detailed details are outlined in Table 1.

**Table 1:** GWAS data sources and information included in the current study.

Phenotype	Sample size	Number of SNPs	Population	Author	Publication time	GWAS ID
*Exposure*						
Educational attainment (college completion)	470,941	11,972,619	Europea n	Loh PR	2018	ebi-a-GCST90 029012
Educational attainment (years of education)	461,457	11,972,619	Europea n	Loh PR	2018	ebi-a-GCST90 029013
Cognitive performance	257,841	10,066,414	Europea n	Lee JJ	2018	ebi-a-GCST00 6572
Intelligence	269,867	9276,181	Europea n	Savage JE	2018	ebi-a-GCST00 6250
Outcome						
Carpal tunnel syndrome	480,201	24,181,062	Europea n	Saka ue S	2021	ebi-a-GCST90 018813
Mediator						
Smoking initiation	607,291	11,802,365	Europea n	Liu M	2019	ieu-b-4877
Smoking	NA	16,379,853	Europea n	NA	2021	finn-b-SMOKI NG
Smoking behavior (cigarettes smoked per day)	4772	8648,224	Europea n	Buch Wald J	2020	ebi-a-GCST00 9968
Alcoholic drinks per week	335,394	11, 887,865	Europea n	Liu, M	2019	ieu-b-73
Time spent watching television (TV)	437,887	9851,867	Europea n	Ben Elswo rth	2018	ukb-b-5192
Time spent using computer	360,895	9851,867	Europea n	Ben Elswo rth	2018	ukb-b-4522
Body mass index (BMI)	454,884	9851,867	Europea n	Ben Elswo rth	2018	ukb-b-2303
Type 1 diabetes	NA	16,380,008	Europea n	NA	2021	finn-b-E4_DM 1
Type 2 diabetes	NA	16,380,440	Europea n	NA	2021	finn-b-E4_DM 2
Depression	484,598	9587,836	NA	Hand an Melike	2021	ebi-a-GCST90 038650
Body fat percentage	331,117	10,894,596	Europea n	Neal e	2017	ukb-a-264
Leisure/social activities: other group activity	461,369	9851,867	Europea n	Ben Elswo rth	2018	ukb-b-4077
Moderate to vigorous physical activity levels	377,234	11,808,007	Europea n	Klime ntidis YC	2018	ebi-a-GCST00 6097
Job involves heavy manual or physical work	263,615	9851,867	Europea n	Ben Elswo rth	2018	ukb-b-2002
Job involves mainly walking or standing	263,556	9851,867	Europea n	Ben Elswo rth	2018	ukb-b-4461
Total cholesterol levels	437,878	4232,052	Europea n	Barto n AR	2021	ebi-a-GCST90 025953
LDL cholesterol	440,546	12,321,875	Europea n	Richa rdson, Tom	2020	ieu-b-110
HDL cholesterol	403,943	12,321,875	Europea n	Richa rdson, Tom	2020	ieu-b-109
Triglycerides	441,016	12,321,875	Europea n	Richa rdson , Tom	2020	ieu-b-111
Total testosterone levels	194,453	16,131,612	Europea n	Ruth KS	2020	ebi-a-GCST90 012113
Serum 25-hydroxyvitamin D levels	417,580	8401,108	Europea n	Reve z JA	2020	ebi-a-GCST90 000617
Non-cancer illness code, self-reported: osteoporosis	462,933	9851,867	Europea n	Ben Elswo rth	2018	ukb-b-12141
Average total household income before tax	397,751	9851,867	Europea n	Ben Elswo rth	2018	ukb-b-7408

GWAS = Genome-Wide Association Study, HDL = high-density lipoprotein, LDL = low-density lipoprotein.

### 2.3. Instrumental variables (IVs)

To ensure that the IVs adequately fulfilled the 3 basic premises of the MR, we implemented rigorous criteria for SNP selection. At the genome-wide level, we selected SNPs that exhibited a significant association with exposure (*P* < 5 *×* 10^-8^). SNPs that exhibited linkage disequilibrium were excluded (*r*^2^ < 0.01 in 10,000 kilobases windows). Furthermore, palindromic SNPs were omitted during the harmonization of educational attainment and CTS GWAS. To address potential biases arising from weak IVs, we calculated F statistics, ensuring that all selected IVs surpassed the threshold F > 10.

### 2.4. MR estimates and sensitivity analysis

The inverse variance weighted (IVW) method was employed as the primary analysis in UVMR, MVRV, and reverse MR. For both UVMR and reverse MR, a fixed effects model was applied when the heterogeneity *P* > .05; conversely, a random-effects model was applied (*P* < .05). Heterogeneity was evaluated using the I^2^ index, Cochran Q statistic, and Rucker Q statistic calculated using the IVW and MR-Egger methods, respectively. MR-Egger and MR pleiotropy residual sum and outlier were used to identify potential horizontal pleiotropy. To ascertain whether any single SNP could affect the cause-and-effect connection between the exposure and the outcome, a “leave-one-out” analysis was performed. Using a 2-step MR analysis, we quantified the influence of educational attainment on CTS (β_all), the effect of educational attainment on health and lifestyle factors (β1), and the effect of health and lifestyle factors on CTS (β2) using UVMR. The mediation effect was obtained by multiplying (β1 × β2), while the mediation proportion was obtained using the formula (β1 × β2)/β_all × 100%.

### 2.5. Statistics

Statistical analyses were conducted using the TwoSampleMR package (version 0.5.7) in the R software(version 4.4.0).

## 3. Results

### 3.1. Causal associations among educational attainment, cognition, intelligence, and CTS

**Figure 3.**
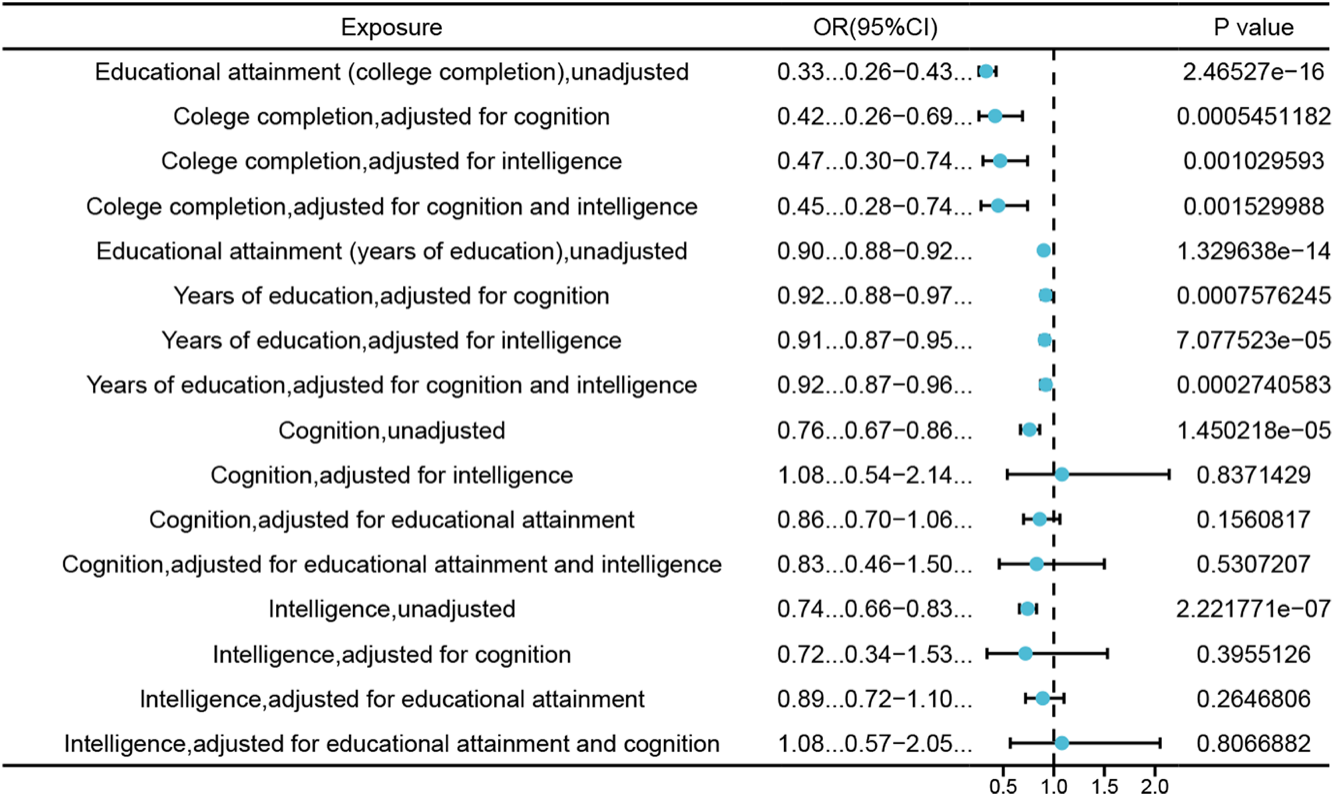
UVMR, MVMR were performed on the exposures (education attainment, cognition and intelligence) and CTS, whether or not adjusted for other exposures. CTS = carpal tunnel syndrome, UVMR = univariable Mendelian randomization, MVMR = multivariable Mendelian randomization.

In our investigation, we employed UVMR analysis to explore the causal relationships among educational attainment (college completion and years of education), cognition, intelligence and CTS. The results indicated that college completion (β = -1.1018, SE = 0.1344, *P* < .05, odds ratio [OR] = 0.33, 95% CI: 0.26–0.43), years of education (β = −0.1049, SE = 0.0136, *P* < .05, OR = 0.90, 95% CI: 0.88–0.92), cognition (β = −0.2803, SE = 0.0647, *P *< .05, OR = 0.76, 95% CI: 0.67–0.86), and intelligence (β = −0.2980, SE = 0.0575, *P* < .05, OR = 0.74, 95% CI: 0.66–0.83) displayed protective properties against the development of CTS (Fig. 3).

The findings derived from the MVMR analysis demonstrated a statistically meaningful causal association between college completion, years of education and CTS, although it was accompanied cognition (β = −0.8706, SE = 0.2518, *P* < .05, OR = 0.42, 95% CI: 0.26–0.69), (β = −0.0787, SE = 0.0234, *P* < .05, OR = 0.92, 95% CI: 0.88–0.97); intelligence (β = −0.7592, SE = 0.2313, *P* < .05, OR = 0.47, 95% CI: 0.30–0.74), (β = −0.0918, SE = 0.0231, *P* < .05, OR = 0.91, 95% CI: 0.87–0.95) or both (β = −0.7939, SE = 0.2505, *P* < .05, OR = 0.45, 95% CI: 0.28–0.74), (β = −0.0880, SE = 0.0242, OR = 0.92, 95% CI: 0.87–0.96). After adjusting for educational attainment and cognitive/intelligence factors, the relationship between cognition and intelligence and CTS lost its statistical significance (Fig. 3).

**Figure 4.**
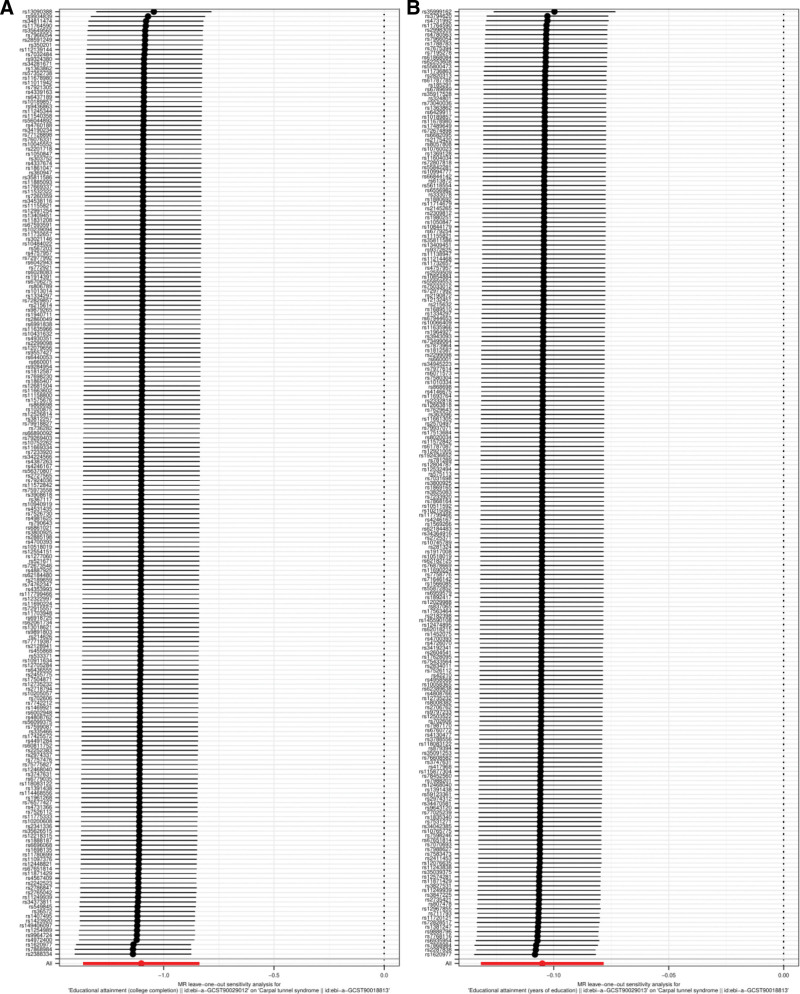
Results of leave-one-out method sensitivity analysis for education attainment on CTS. (A) College completion on CTS. (B) Years of education on CTS. CTS = carpal tunnel syndrome.

Additionally, MR analysis revealed no significant heterogeneity between college completion and CTS (IVW, *P* = .054, I^2^ = 14.22%; MR-Egger, *P* = .058, I^2^ = 13.99%). In contrast, significant heterogeneity was observed between years of education and CTS (IVW, *P* < .05, I^2^ = 29.91%; MR-Egger, *P* < .05, I^2^ = 30.23%), necessitating the application of a random-effects IVW (Table 2). No evidence of pleiotropy was detected by the MR-Egger test for college completion, years of education, and CTS (*P* = .218, *P* = .797) (Table 2). The “leave-one-out” analysis confirmed that no individual SNP had a substantial impact on overall causal association estimation in either MR analysis, thereby reinforcing the robustness of the study’s findings (Fig. 4).

**Table 2:** The results of heterogeneity and horizontal pleiotropy of educational attainment and CTS in the MR analysis.

		Heterogenity test		Pleiotropy test
Exposure	Outcome	Cochran Q test	Rucher Q test	Egger intercept
		(*P* value)	(*P* value)	(*P* value)
		IVW	MR-Egger	MR-Egger
College completion	CTS	.054	.058	.218
Years of education	CTS	<.001	<.001	.797

CTS = carpal tunnel syndrome, MR = Mendelian randomization, IVW = inverse variance weighted.

### 3.2. A reverse MR analysis

We established a causal role for educational attainment in CTS. Subsequently, we performed a reverse MR analysis, which revealed the absence of a significant causal effect of CTS on educational attainment (college completion β = −0.0044, SE = 0.0085, *P* = .61; years of education β = −0.0241, SE = 0.0748, *P* = .75) (Table 3).

**Table 3:** The results of reverse MR between educational attainment and CTS.

Exposure	Outcome	Method	Nsnp	β	SE	*P*
CTS	College completion	IVW	10	-0.0044	0.0085	.61
CTS	Years of education	IVW	10	-0.0241	0.0748	.75

MR = Mendelian randomization, CTS = carpal tunnel syndrome, IVW = inverse variance weighted.

### 3.3. Impacting of educational attainment on potential mediating factors

**Figure 5.**
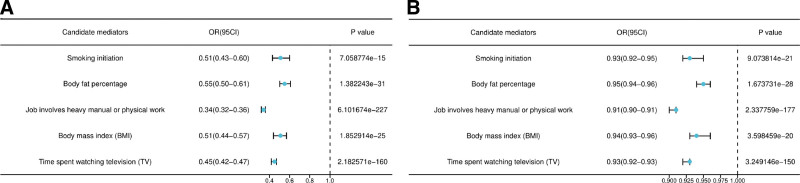
Effect of education attainment on mediator. (A) Effect of college completion on mediator. (B) Effect of years of education on mediator.

In our study, we identified 24 potential mediators and conducted UVMR analysis to explore the associations between educational attainment and these mediators, in addition to the relationships between mediators and CTS. Through this analysis, we identify 5 mediators that demonstrate significant effects. The UVMR analysis indicated that both college completion and years of education serve as protective factors against the following mediators: smoking initiation (β = −0.6737, SE = 0.8655, *P* < .05, OR = 0.51, 95% CI: 0.43–0.60; β = −0.0691, SE = 0.0074, *P* < .05, OR = 0.93, 95% CI: 0.92–0.95); body fat percentage (β = −0.5900, SE = 0.0505, *P* < .05, OR = 0.55, 95% CI: 0.50–0.61; β = −0.0501, SE = 0.0045, *P* < .05, OR = 0.95, 95% CI: 0.94–0.96); job involving heavy manual or physical work (β = −1.0826, SE = 0.0337, *P* < .05, OR = 0.34, 95% CI: 0.32–0.36; β = −0.0957, SE = 0.0034, *P* < .05, OR = 0.91, 95% CI: 0.90–0.91); BMI (β = −0.6825, SE = 0.0654, *P* < .05, OR = 0.51, 95% CI: 0.44–0.57; β = −0.0574, SE = 0.0062, *P* < .05, OR = 0.94,95% CI: 0.93–0.96); time spending watching TV (β = −0.8073, SE = 0.0299, *P* < .05, OR = 0.45, 95% CI: 0.42–0.75; β = −0.0745, SE = 0.0029, *P* < .05, OR = 0.93, 95% CI: 0.92–0.93) (Fig. 5).

### 3.4. Impacting of potential mediating variables on CTS

**Figure 6.**
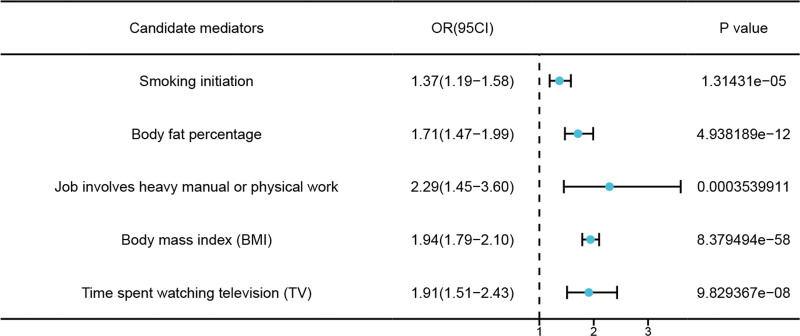
Effect of mediator on CTS. CTS = carpal tunnel syndrome.

UVMR show 5 mediators as risk factor of CTS: smoking initiation (β = 0.3142, SE = 0.0721, *P* < .05, OR = 1.37, 95% CI: 1.19–1.58), body fat percentage (β = 0.5376, SE = 0.0778, *P* < .05, OR = 1.71 95% CI: 1.47–1.99), job involves heavy manual work (β = 0.8270, SE = 0.2315, *P* < .05, OR = 2.29, 95% CI: 1.45–3.60), BMI (β = 0.6627, SE = 0.0414, *P* < .05, OR = 1.94, 95% CI: 1.79–2.10), time spent watching TV (β = 0.6483, SE = 0.1216, *P* < .05, OR = 1.91, 95% CI: 1.51–2.43) (Fig. 6).

### 3.5. Mediation effect (95% CI) and mediation proportion (%) of mediating factors between educational attainment and CTS

**Figure 7.**
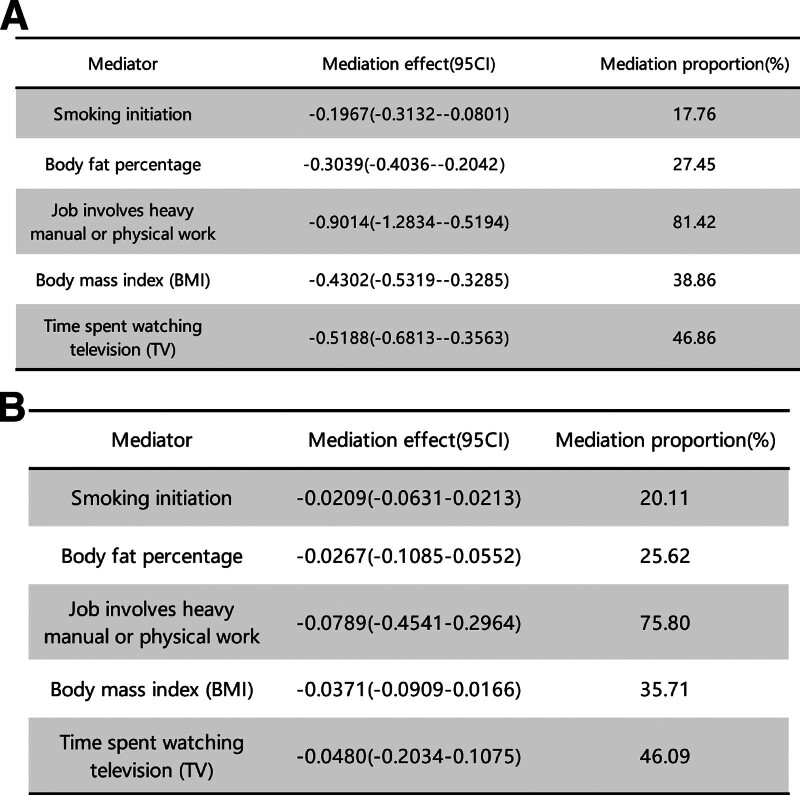
Mediation effect (95% CI) and mediation proportion (%) of mediating factors between education attainment and CTS. (A) Mediation effect (95% CI) and mediation proportion (%) of mediating factors between college completion and CTS. (B) Mediation effect (95% CI) and mediation proportion (%) of mediating factors between years of education and CTS. CTS = carpal tunnel syndrome.

We calculated the mediation effects (95% CI) and proportion (%) of mediators between college completion and years of education and CTS using 2-step MR. The results showed smoking initiation: mediation effect = −0.1967 (−0.3132 to −0.0801), mediation proportion (17.76%); mediation effect = −0.0209 (−0.0631 to 0.02310), mediation proportion (20.11%); body fat percentage: mediation effect = −0.3039 (−0.4036 to −0.2042), mediation proportion (27.45%); mediation effect = −0.0267 (−0.1085 to 0.0552), mediation proportion (25.62%); job involving heavy manual or physical work: mediation effect = −0.9014 (−1.2834 to −0.5194), mediation proportion (81.42%); mediation effect = −0.0789 (−0.4541 to 0.29640), mediation proportion (75.80%); BMI: mediation effect = −0.4302 (−0.5319 to −0.3285), mediation proportion (38.86%); mediation effect = −0.0371 (−0.0909 to 0.0166), mediation proportion (35.71%); time spent watching TV: mediation effect = −0.5188 (−0.6813 to −0.3563), mediation proportion (46.86%); mediation effect = −0.0480 (−0.2034 to 0.1075), mediation proportion (46.09%) (Fig. 7).

## 4. Discussion

CTS is a prevalent condition that adversely affects an individual’ health and daily activities, restricts work capacity, and imposes an economic burden on both patients and society.^[13]^ Historically, research into the risk factors of CTS has predominantly adopted a biomedical perspective, emphasizing the working environment, sex, and comorbid conditions.^[10,14,15]^ Standard therapeutic interventions include rest, oral nonsteroidal anti-inflammatory drugs, corticosteroid injections, and surgery.^[16]^ In fact, the high incidence of CTS and disease progression remain significant challenges for effective disease management. Research has demonstrated that higher levels of educational attainment serves as protective factor against chronic musculoskeletal disorders.^[17]^ Previous epidemiological research has indicated that individuals with lower educational attainment have a higher risk of developing CTS and tend to have poorer prognosis.^[18]^ To minimize the confounding factors inherent in observational studies, we conducted UVMR, MVRV, reverse MR, and 2-step MR to examine the causal relationships between educational attainment and CTS.

Established phenotypic and genetic interrelations among education, cognition, and intelligence. We initially tested the correlations among educational attainment, cognition, intelligence and CTS using UVMR. Significant genetic correlations were observed between educational attainment, cognition, intelligence, and CTS. However, when the 3 variables were adjusted for each other in MVMR, only educational attainment demonstrated an independent causal association with CTS. Additionally, the results of reverse MR indicated that the relationship between educational attainment and CTS is unidirectional. This indicates that enhancing educational level may facilitate the implementation of preventive strategies for CTS.

A significant discovery of this study was the recognition and measurement of the mediating influence exerted by several health and lifestyle elements on the association between educational achievement and CTS. In particular, smoking initiation, time spent watching TV, body fat percentage and job involving heavy manual or physical work were found to mediate the relationship between educational attainment and CTS.

Epidemiological studies have identified occupational exposure as a major risk factor, such as repetitive movements of the wrist and hand, heavy manual labor, and maintenance of special wrist and hand postures.^[3,10]^ This MR shows that educational attainment influences the incidence of CTS through occupations that require heavy manual or physical exertion. However, the mediating effect of jobs primarily involving walking or standing was insignificant in this process. This phenomenon can attributed to 2 factors. First, educational attainment is a crucial determinant of occupational type. Lower educational levels are associated with manual labor. Second, individuals with lower educational attainment often exhibit a lack of health awareness, and MR results show that this process is not related to family income. Consequently, these individuals face an increased likelihood of developing CTS; however, they frequently overlook the implementation of protective strategies. Individuals who develop CTS often exhibit deficiencies in self-care knowledge and skills.^[19]^ When symptoms are mild, patients may not seek medical assistance. Even if symptoms are exacerbated, adherence to medical advice may remain insufficient. Delays in diagnosis and treatment frequently lead to poor patient outcomes.^[20]^ This phenomenon may explain why individuals with lower educational attainment tend to experience more severe symptoms and worse prognosis. These findings suggest that to fundamentally reduce the incidence of CTS and improve therapeutic effects, efforts should be directed towards increasing awareness of self-protection and improving self-care skills among high-risk individuals, regardless of their income level.

Smoking initiation, body fat percentage, BMI, and time spent watching TV also played mediating roles in the relationship between educational attainment and CTS. These mediators were related to personal lifestyle habits. Previous studies have identified smoking as a risk factor for CTS, particularly heavy smoking during adolescence.^[21]^ This association may be attributed to smoking-induced vascular damage to the median nerve, leading to nerve degeneration and fibrosis, which ultimately results in CTS.^[22]^ However, in this study, only smoking initiation exhibited a mediating effect on the relationship between educational attainment and CTS. This indicates that smoking can significantly contribute to the onset of CTS. The observed relationship may be due to the lower occurrence of smoking among individuals with higher levels of education, who are generally more cognizant of the health hazards associated with tobacco use and, as a result, may opt to refrain from smoking. Prior research has demonstrated that an extended duration of education correlates with a reduced risk of smoking and heightened probability of cessation.^[23]^ Additionally, BMI and obesity were found to be positively correlated with CTS, while sedentary lifestyle and poor eating habits were considered risk factors for the development of obesity.^[24–26]^ While the influence of leisure or social activities, levels of physical activity, diabetes, and cholesterol were not substantiated, this MR study affirms that body fat percentage, BMI, and duration of TV viewing are significant risk factors for CTS, which is consistent with findings from earlier research. Conversely, weight loss can help reduce the incidence of CTS and delay its progression.^[27]^ Higher educational attainment is generally associated with healthier lifestyles.^[28]^ Individuals with higher educational attainment tend to consume more fruit and vegetables and are more willing to engage in physical exercise during their leisure time.^[29]^ Thus, avoiding sedentary behavior and reducing fat accumulation may help lower the incidence of CTS.

This study had several strengths. The SNPs employed as IVs for MR effectively mitigated bias due to confounding factors and prevented reverse causation bias. The results of the sensitivity analyses were consistent across the 2 phenotypes of educational attainment. Furthermore, educational attainment was found to have an independent causal relationship with CTS, underscoring the robustness of our results.

However, this study had several limitations. First, summary statistics were obtained from GWAS data, precluding the possibility of conducting subgroup analysis. Second, the data used were exclusively from European populations, potentially constraining the applicability of the findings to broader populations. Furthermore, although our study concentrated on CTS, the generalizability of these findings to the types and etiologies of CTS remains to be determined.

## 5. Conclusion

The conclusion drawn from this study indicates a negative association between educational attainment and the incidence of CTS. The key mediating factors identified include occupations that require substantial manual or physical labor, initiation of smoking, body fat percentage, BMI, and duration of TV viewing. Therefore, it is imperative to target individuals with lower educational attainment, providing them with the necessary knowledge and skills to decrease the incidence of CTS and mitigate its symptoms. Furthermore, the promotion of healthy lifestyles is vital within this framework.

## Author contributions

**Conceptualization:** Weiquan Hu, Xianping Huang, Qinfei Zhao.

**Formal analysis:** Qinglin Liu.

**Methodology:** Yunrong Lai, Suping Hu.

**Writing – original draft:** Weiquan Hu.

**Writing – review & editing:** Weiquan Hu.
